# Supplementary material for: Different Putative Methyltransferases Have Different Effects on the Expression Patterns of Cellulolytic Genes
Source: J Fungi (Basel). 2023 Nov 17;9(11):1118. doi: 10.3390/jof9111118 (PMC10671955; doi:10.3390/jof9111118)
Supplement: Supplementary file 1 [file jof-09-01118-s001.zip › Table S1.docx]

**Table S1** Primers used in this study

| **Primers** | **Primer sequences (5'-3')** |
| --- | --- |
| Primers for construction of Δ*mtr23C* strain | |
| mtr23C-UF | GTGAGATCGATTGGTGCACGCATTCG |
| mtr23C-UR | GTCTGCGGCGCGTTCTCGAGGAAGTTGCCGTGGGCCTATCTGGATGATATACTGC |
| mtr23C-DF | GGAAATGTGTAACGGTATTGACTAAAAGGGGGGCTAGTCATACCTCTGTGCCTGG |
| mtr23C-DR | GAGGTCGGAATCTTGAGCACAGTCAC |
| mtr23C-NF | GTGTTCGAAGATTGTCATCTGAAAGGC |
| mtr23C-NR | CGCTTAAGGAGAGATTGCGGAGTTC |
| ptrA-F | GGGCAATTGATTACGGGATCCCATTG |
| ptrA-R | ATGGGGTGACGATGAGCCGCTCTTGC |
| Primers for construction of Δ*mtr23D* strain | |
| mtr23D-UF | GTCAAGAGGCATCACTTCCGTTTG |
| mtr23D-UR | GTTACCAATGGGATCCCGTAATCAATTGCCCGCAAAAGTGGTGATCCACAAACGTATC |
| mtr23D-DF | GATGCAAGAGCGGCTCATCGTCACCCCATCATGTCGCAGTTTGGCGGTTGATCC |
| mtr23D-DR | CATACCTTCCACTTGTCAATGTC |
| mtr23D-NF | GTGCTGTCACGTATGTCAGGAG |
| mtr23D-NR | GCCTTCAAATTAAGGCACACATTCC |
| Primers for construction of Δ*mtr23E* strain | |
| mtr23E-UF | CAATTAGCCGACCTTTGAGGCTGG |
| mtr23E-UR | GTTACCAATGGGATCCCGTAATCAATTGCCCTTTCCTTCAATGCTTGATAC |
| mtr23E-DF | GATGCAAGAGCGGCTCATCGTCACCCCATGTCTTTCCAAACCCCCCTC |
| mtr23E-DR | CTGAAGATTCACTTGGCGGGCCTAC |
| mtr23E-NF | CTGTTCTGTGGAGCGGGTTATCG |
| mtr23E-NR | GCTGCACCCGTCCTGCAAACCTGTC |
| Primers for construction of Δ*mtr23F* strain | |
| mtr23F-UF | GACCCACCAACATCGCACAAGGG |
| mtr23F-UR | GTTACCAATGGGATCCCGTAATCAATTGCCCGGTTTTACGATGCAGGC |
| mtr23F-DF | GCGGCTCATCGTCACCCCATAGCGGCATGGGGACCTTGATTCAACTG |
| mtr23F-DR | CGGCACTCTGCGGTAAGAAGTAGTTG |
| mtr23F-NF | GCTATTTGTCCCTACCCCGTGATC |
| mtr23F-NR | GGAGAGTTGCGCTTGTTTCGCTC |
| Primers for construction of Δ*mtr23G* strain | |
| mtr23G-UF | AGTCAGGCGAATGGTCGGGTG |
| mtr23G-UR | GATCCCGTAATCAATTGCCCGGCCCCCCCGTCAGAGAACAGG |
| mtr23G-DF | GCGGCTCATCGTCACCCCATTCACAGTGGGACAGGGGTCATC |
| mtr23G-DR | GATGACTGGGCCAGAGCGCATCGC |
| mtr23G-NF | GCCAACCCAGGCCGGGACACG |
| mtr23G-NR | GATAGGCCCATGTCAATGGTCCG |
| Primers for construction of Δ*mtr23H* strain | |
| mtr23H-UF | GAGCCTGTGGGGAGCATAGAGCCC |
| mtr23H-UR | GATCCCGTAATCAATTGCCCGTGATGTTGCAGGTCAAGGCGATC |
| mtr23H-DF | GCGGCTCATCGTCACCCCATGCTCGATACGGATTTTGCAAGATG |
| mtr23H-DR | GCGCCACATTCAGGATGGAGTTG |
| mtr23H-NF | CAACCTGAGTATTCACCGCAGG |
| mtr23H-NR | GAAGTTCAGTAAGCCAGACGTTCAC |
| Primers for construction of Δ*mtr25A* strain | |
| mtr25A-UF | GGATCAGGTTTCGACTTCCCATTGGC |
| mtr25A-UR | GTCTGCGGCGCGTTCTCGAGGAAGTTGCGCTGGCTGATCGTTTCATGGATGCATTTAACG |
| mtr25A-DF | GGAAATGTGTAACGGTATTGACTAAAAGGGGCCATCTATTCTTCTCATGTAGACTATG |
| mtr25A-DR | GGCATACGCGATCAGATCATTCACCG |
| mtr25A-NF | GGCTGTCAGGGTCTTTCTGCAATTCG |
| mtr25A-NR | GTGGTCCTCTTCAACTAGCGACTGG |
| Primers for construction of Δ*mtr25B* strain | |
| mtr25B-UF | GGATCTTCTTTGACAAGAGAACGACAC |
| mtr25B-UR | GTTACCAATGGGATCCCGTAATCAATTGCCCGACTAATTTCGATAAGGGGTTGGAG |
| mtr25B-DF | GATGCAAGAGCGGCTCATCGTCACCCCATTGCCTGGCCTACCTAGAGCCACGAGATAC |
| mtr25B-DR | CCTTGCCTGAGATCGACGATAATGCG |
| mtr25B-NF | CGTCAAGAGCTTCGGTCTGGATGGAC |
| mtr25B-NR | GCCATCTTGCATACTTGCTGCAGCG |
| Primers for Southern Blot | |
| Soumtr23C-F | GGGCTAGTCATACCTCTGTGCCTG |
| Soumtr23C-R | GCTTAAATTGCTTCTGACCGTTC |
| Soumtr23D-F | CATGTCGCAGTTTGGCGGTTGATC |
| Soumtr23D-R | CTGCCAAACTGTGGCAAATCCGC |
| Soumtr23E-F | GTCTTTCCAAACCCCCCTCTCAC |
| Soumtr23E-R | GCCGTCAATTGCGATTCCAAG |
| Soumtr23F-F | AGCGGCATGGGGACCTTGATTC |
| Soumtr23F-R | GTCGAAGAAGAGTCGAACGTCC |
| Soumtr23G-F | TCACAGTGGGACAGGGGTCATCG |
| Soumtr23G-R | CATACTTGAGAGTATAATCGGAG |
| Soumtr23H-F | GCTCGATACGGATTTTGCAAGATG |
| Soumtr23H-R | TCTCTCCGGCTGTCTTTTCTACAG |
| Soumtr25A-F | TTCGCCTGGTGGTGCAGCTGCG |
| Soumtr25A-R | TTCTTAGGTTGGAAATGAAAACC |
| Soumtr25B-F | TGCCTGGCCTACCTAGAGCCACG |
| Soumtr25B-R | TGAAAAGGGCTTGGAGCATCGG |
| Primers for qPCR | |
| q23C-F | CGCCCTGGCCTCGAGACCCG |
| q23C-R | GTTTGGACATATGCGTGCAAC |
| q23D-F | GATCCCAGAATGCGGGAGATAG |
| q23D-R | CATAGGGAAATATGCCTTCAAC |
| q23E-F | CCCAAGGATAAGGTCCTCAAG |
| q23E-R | TCACGCGTATCCGTATCCATG |
| q23F-F | CTCTGCTCATTATTGATGGCAG |
| q23F-R | GTAATGACGCGCTGTGATTCGTG |
| q23G-F | CGGCCGCGTTGGCAAAGTCAC |
| q23G-R | GACTTGTGTCGAGCGGCTTTTG |
| q23H-F | GAATTGGCTGGAAGCGCTGGC |
| q23H-R | TCAAGGCTGTTCCCTCCTTGCG |
| q25A-F | GAGAACCATGCGGCGATCGTC |
| q25A-R | TCACTTCTTCACTGCAATGAC |
| q25B-F | GATGAGCTCAATCTGACTATTAC |
| q25B-R | CTACAACGGTTTCTGTCCAATC |
| CBH-QF | CCACCACCACTACCAGCAAGG |
| CBH-QR | GTAGCCAACACCACCGCACT |
| EG-QF | ACCGCTGCTCAGACCACGAC |
| EG-QF | TGGGTCCCGAGTAGCCAACG |
| XYN-QF | GGTCTCCAGGCTCACTTCATC |
| XYN-QR | GTCGAGGGCAAGTTCATACG |
| xlnR-QF | CCTGTGGATTTCCTCCGATTC |
| xlnR-QR | CCGTCAGAAGAAGCAGGAAGC |
| creA-QF | TGGGTACGAGTGAACTCCATCTT |
| creA-QR | TGTGACCTTGACCAGGACTGTAA |
| ACT-QF | GTTCCATTCTCGCCTCCCTCT |
| ACT-QR | AGAAGCACTTGCGGTGAACGA |
| Primers for construction of Mtr25A-GFP strain | |
| GFP-UF | CTGTCATGATTTCGAGTCGGGCC |
| GFP-UR | CAGCTCCTCGCCCTTGCTCACCATTCCTCCTCCTCCCTTCTTCACTGCAATGACGAAG |
| GFP-F | ATGGTGAGCAAGGGCGAGGAGCTG |
| GFP-R | TTACTTGTACAGCTCGTCCATGCCG |
| GFP-MF | ATGGTGAGCAAGGGCGAGGAGCTG |
| GFP-MR | TTACTTGTACAGCTCGTCCATGCCG |
| GFP-DF | CCGTCACCAGCCCCTGGGTTGTTCGCCTGGTGGTGCAGCTGCGC |
| GFP-DR | GAAGAATCTCGCGACGAGGATG |
| GFP-NF | GTCATTCACATCAAGGACGGAGG |
| GFP-NR | CATCCTCGTCGCGAGATTCTTC |
| Primers for construction of Mtr25A-TAP strain | |
| TAP-UF | CGAAGGTCGTGATAAAGTGTTTGCGC |
| TAP-UR | GACGTCGTAAGGGTACTTGTCATCGTCGTCCTTGTAGTCCTTCTTCACTGCAATGACGAAGAGTC |
| TAP-MF | GGACGACGATGACAAGTACCCTTACGACGTCCCCGACTACGCCTGAGGGCAATTGATTACGGGATCCCATTGG |
| TAP-MR | GATGGCGCAGCTGCACCACCAGGCGAAATGGGGTGACGATGAGCCGCTCTTGCATC |
| TAP-DF | GATGCAAGAGCGGCTCATCGTCACCCCATTTCGCCTGGTGGTGCAGCTGCGCCATC |
| TAP-DR | GGCATACGCGATCAGATCATTCACCG |
| TAP-NF | GTCATTCATTGCGGAGCGCCATTACG |
| TAP-NR | GACCACTGGATTCAAATCATTGAAGC |
