# Supplementary figures and images for: Different Putative Methyltransferases Have Different Effects on the Expression Patterns of Cellulolytic Genes
Source: J Fungi (Basel). 2023 Nov 17;9(11):1118. doi: 10.3390/jof9111118 (PMC10671955; doi:10.3390/jof9111118)

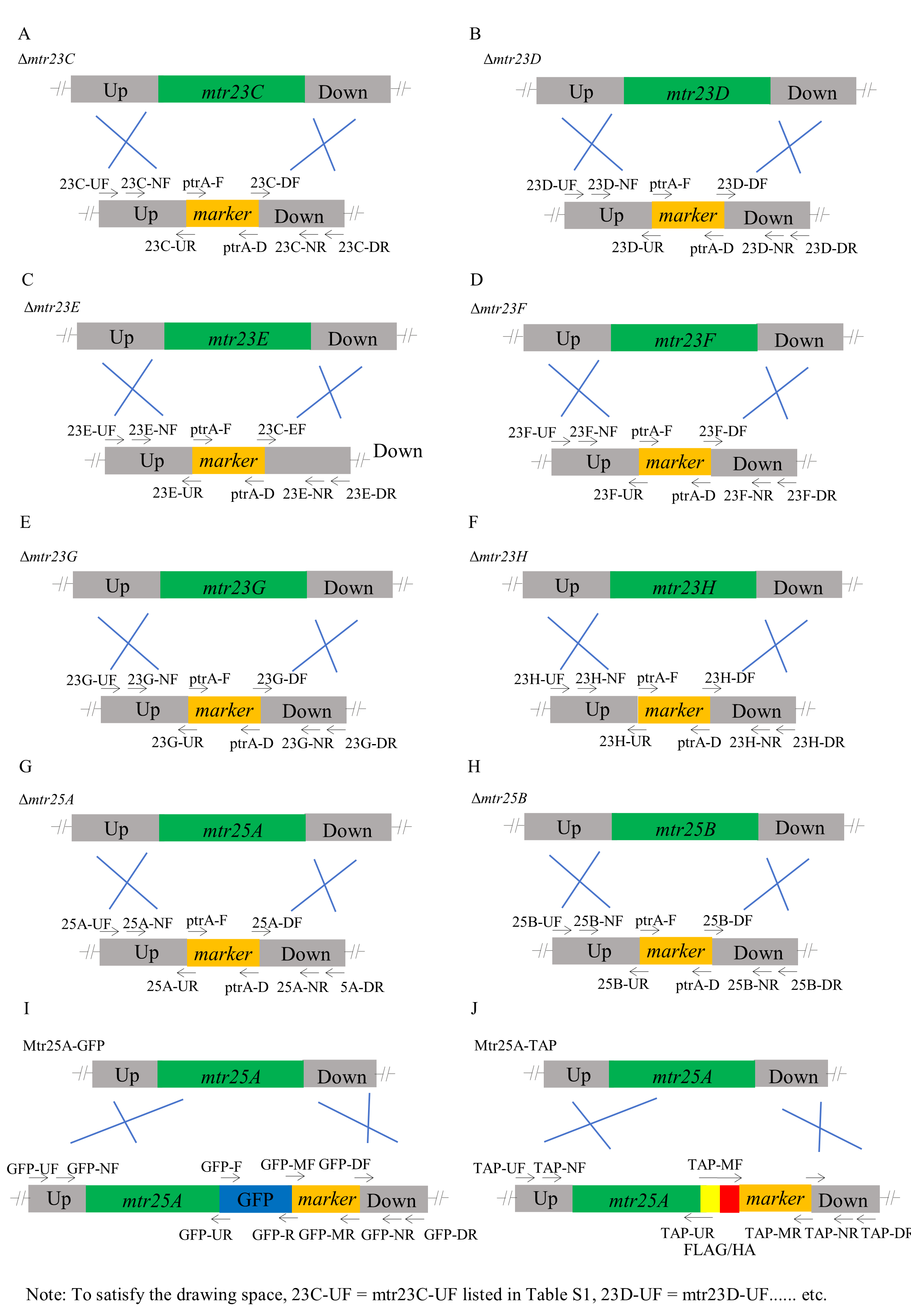

Supplement: Supplementary file 1 [file jof-09-01118-s001.zip › Figure S1.tif]

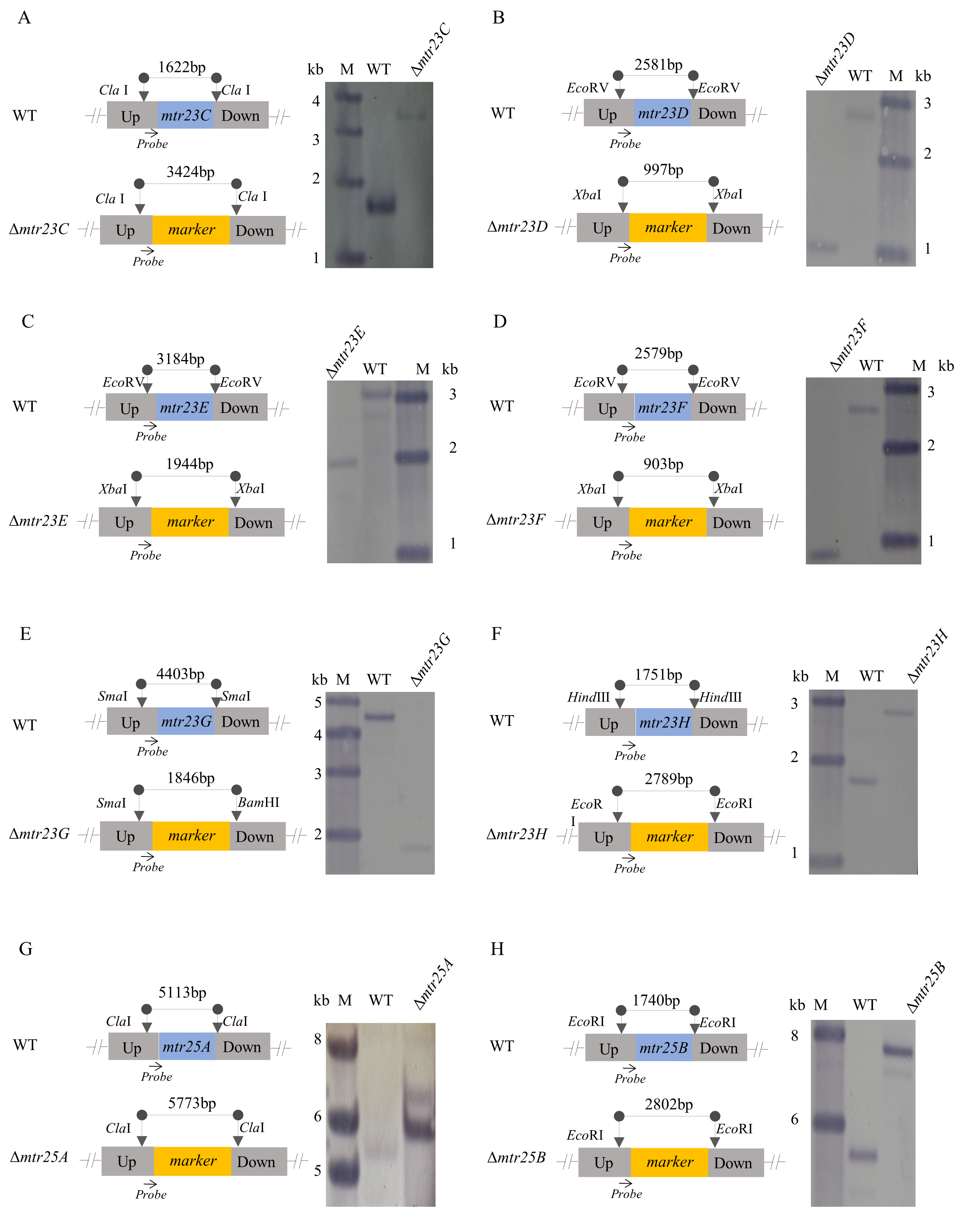

Supplement: Supplementary file 1 [file jof-09-01118-s001.zip › Figure S2.tif]
